# Natural Antibacterial and Antivirulence Alkaloids From *Macleaya cordata* Against Methicillin-Resistant *Staphylococcus aureus*

**DOI:** 10.3389/fphar.2022.813172

**Published:** 2022-03-17

**Authors:** Zhi-Hai Liu, Wei-Mei Wang, Zhen Zhang, Liang Sun, Shuai-Cheng Wu

**Affiliations:** ^1^ College of Veterinary Medicine, Qingdao Agricultural University, Qingdao, China; ^2^ College of Chemistry and Pharmaceutical Sciences, Qingdao Agricultural University, Qingdao, China; ^3^ Beijing Advanced Innovation Center for Food Nutrition and Human Health, College of Veterinary Medicine, China Agricultural University, Beijing, China

**Keywords:** 6-ethoxysanguinarine, MRSA, antibacterial activity, antivirulence activity, host immunomodulatory activity

## Abstract

The emergence and spread of antibiotic-resistant bacteria, such as methicillin-resistant *Staphylococcus aureus* (MRSA), underly the urgent need to develop novel antibacterial drugs. *Macleaya cordata*, a traditional medicinal plant, has been widely used in livestock animals, plants, and humans. Alkaloids are the primary bioactive compounds of *Macleaya cordata* and exhibit antibacterial, antiinflammatory, and antioxidant activities. Nevertheless, the antibacterial compounds and mode of action of *Macleaya cordata* remain unclear*.* In the present study, we investigated the antibacterial activity and mode of action of alkaloids from *Macleaya cordata*. Sanguinarine, 6-ethoxysanguinarine (6-ES), 6-methoxydihydrosanguinarine (6-MS), chelerythrine (CH), and dihydrochelerythrine (DICH) exhibited good antibacterial activity against Gram-positive bacteria, including MRSA. 6-ES rapidly killed MRSA, possibly by interfering with membrane and metabolic functions including ROS production by targeting the membrane and FtsZ in *S. aureus*. Additionally, 6-ES directly suppressed the hemolytic activity of *α*-hemolysin, alleviated inflammatory responses, and eliminated intracellular MRSA, as well as displayed low development of drug resistance, *in vitro*. Finally, a 6-ES-loaded thermosensitive hydrogel promoted wound healing in mice infected with MRSA. These results supported 6-ES as a novel potential candidate or leading compound with antibacterial, antivirulence, and host immunomodulatory activities in fighting against bacterial infections.

## Introduction

The emergence and spread of antibiotic-resistant bacteria, such as methicillin-resistant *Staphylococcus aureus* (MRSA), pose a severe threat to public healthcare (Chen et al., 2016; Song et al., 2020). A critical approach to the problem is developing new antibacterial drugs or alternative strategies. Natural products from medicinal plants with chemical diversity are important sources for the discovery and development of antibacterial drugs. Alkaloids are important bioactive components of many medicinal plants and possess diverse pharmacological activities, such as antimicrobial, anti-inflammatory, and antioxidant activities (Kosina et al., 2010; Talman et al., 2019). Thus, alkaloids from medicine plant may *provide new candidate or leading compound for the development of antibacterial drugs.*

*Macleaya cordata (Willd.) R. Br. (Papaveraecae)* is a traditional medicine plant used for dispelling wind, detoxication, elimination of dampness, and relieving pain, and has been used for the treatment of carbuncle, rheumatoid arthritis, wound infection. Alkaloids such as sanguinarine and chelerythrine are the main active components in *Macleaya cordata*, and exhibits antitumor, antioxidant, antibacterial, and antiviral activities (Khin et al., 2018). The extracts of *Macleaya cordata* protected mice challenged with enterotoxigenic *Escherichia coli* (Guan et al., 2019). It has been widely used as a food additive to prevent bacterial diseases in livestock animals (Li et al., 2018). Interestingly, the exacts of *Macleaya cordata* also have been used as plant pesticides to prevent bacterial and insect-associated diseases in vegetable production (Ke et al., 2017; Yan et al., 2021). However, the potential antibacterial compounds and modes of action of *M. cordata* remain unclear.

Plant alkaloids exhibits antibacterial effects *via* multiple antibacterial mechanism, such as inhibition of cell division, increased permeability of the bacterial membrane, and inhibition of bacterial metabolism. Studies also have showed that natural products from medicine plants protected against bacterial infection via modulation host response and bacterial virulence (Wu et al., 2018). Herein, we aimed to investigate the antibacterial activity and mode of action of alkaloids from M. cordata, *thereby provide new candidate or leading compounds for the development of antibacterial drugs.*

## Materials and Methods

### Chemicals

Sanguinarine (SA), 6-ethoxysanguinarine (6-ES), 6-methoxydihydrosanguinarine (6-MS), chelerythrine (CH), and dihydrochelerythrine (DICH) were purchased from Chengdu Biopurify Phytochemicals Ltd. (Chengdu, China). Poloxamer 407 (P407) was purchased from BASF (Ludwigshafen, Germany). 3,3′-Dipropylthiadicarbocyanine iodide (DISC3 (5)) was purchased from Aladdin (Shanghai, China). Peptidoglycan and lipoteichoic acid were purchased from Sigma-Aldrich (St. Louis, United States). SYTO™ nine Green Fluorescent Nucleic Acid Stain (SYTO 9) was purchased from Thermo Fisher (Waltham, United States). Phosphatidylglycerol (PG), cardiolipin (CAL), and lysyl-phosphatidylglycerol (lysyl-PG) were purchased from Avanti Polar Lipids, Inc. (Alabaster, United States). Filamentous temperature-sensitive protein Z (FtsZ) was purchased from Cytoskeleton Inc. (Denver, United States). Propidium iodide (PI), dichlorodihydrofluorescein diacetate (DCFH-DA), and enhanced ATP assay kits were purchased from Beyotime (Shanghai, China).

### Minimum Inhibitory Concentration (MIC)

MICs were determined with the broth microdilution method based on the guide of Clinical and Laboratory Standards Institute. Methicillin-sensitive *Staphylococcus aureus* (MSSA) ATCC29213 and *Escherichia coli* ATCC25922 were purchased from the American Type Culture Collection. MRSA T144, *E. coli* B2*,* and other bacterial strains were donated by Professor Kui Zhu, China Agricultural University. MIC_20_, the minimal inhibitory concentration at which the growth of 20% strains are inhibited. MIC_50_, the minimal inhibitory concentration at which the growth of 50% strains are inhibited. MIC_90_, the minimal inhibitory concentration at which the growth of 90% strains are inhibited. To screen the possible targets of 6-ES, the MICs of 6-ES against MRSA T144 in the presence of bacterial wall and membrane components were measured.

### Time Killing Assay

MRSA T144 was cultured in MHB broth obtain approximately 10^6^ colony-forming units (CFU)/mL, then treated with different concentrations of 6-ES and vancomycin. After incubating MRSA T144 with 6-ES and vancomycin for 0, 1, 3, 6 h, samples were removed with mueller-hinton Agar (MHA) plates, and the number of surviving bacteria was counted. To confirm determine the antibacterial effect of 6-ES depends on metabolism, its bactericidal effect was measured at 0 and 37°C.

### Membrane Function Assay

To investigate effects of 6-ES on the proton motive force (PMF), membrane permeability, and ROS, DISC3 (5), PI, or DCFH-DA were used respectively. Overnight cultures of MRSA T144 were washed 3 times with 5 mM HEPES and 5 mM glucose, pH 7.2. Subsequently, the bacterial cells were incubated with DISC3(5) (1 μM), PI (7.5 μg/ml), or DCFH-DA (10 μM) for 10–30 min. Then, the bacterial cells were treated with 6-ES (0–16 μg/ml), Vancomycin (8 μg/ml), or lysostaphin (8 μg/ml). The fluorescence intensity was measured with 622 nm excitation and 670 nm emission filters for DISC3 (5), 535 nm excitation and 615 nm emission filters for PI, or 488 nm excitation and 525 nm emission filters for DCFH-DA.

### ATP Assay

The bacteria cultured overnight were washed 3 times with PBS, mixed with PBS buffer solution to 0.5 McTurbidiol, and incubated with 6-ES (0–16 μg/ml) for 60 min. Then, the bacteria were collected by centrifugation and the supernatant was used to detect the extracellular ATP content of the bacteria. The precipitation was treated with lysostaphin to detect the content of ATP in bacteria.

### Cytotoxicity Assays

Vero cells or RAW264.7 cells were grown to 70–80% and cultured to 10^5^ Cells/mL in a fresh DMEM containing 2% FBS. Then were seeded in 96-well plates and then cultured wit 6-ES (0–4 μg/ml) for 24 h at 37°C. After incubation for 24 h, Vero cells were washed with PBS and then incubated with WST-1 for 30 min. The cell viability was measured at OD 450 nm. The cell viability of Vero cells without treatment was set at 100%.

### Cytokine Measurement

RAW264.7 cells were infected with heat-killed MRSA T144 cells at a multiplicity of infection (MOI) of 1, and co-cultured with 6-ES (0–0.5 μg/ml) or dexamethasone (DEX, 0.5 μg/ml) for 24 h at 37°C. After incubation for 24 h, the culture supernatants were harvested to measure TNFα levels with ELISA kits (Liu et al., 2020).

### Hemolytic Analysis

MRSA T144 cells were cultured with 6-ES (0–128 μg/ml) for 12 h at 37°C. The supernatants were harvested to detect the hemolytic activity of toxin from *S. aureus* on sheep red blood cells. In brief, 5% sheep red blood cells were cultured with the supernatants for 1 h at 37°C. After incubation for 1 h, the supernatants of sheep red blood cells were harvested by centrifugation, and OD at 570 nm was measured. To evaluate whether 6-ES directly affects the hemolytic activity of *α*-hemolysin from *S. aureus*, sheep red blood cells were treated with 6-ES with or without the supernatants from DMSO-treated MRSA T144.

### Intracellular Bacteria Determination

Vero cells were infected with MRSA T144 at an MOI of 10 and then cultured with 6-ES (0.06–0.5 μg/ml) or vancomycin (8 μg/ml) for 6 h at 37°C in 5% CO_2_. Extracellular bacteria were removed by vancomycin (50 μg/ml), incubated for 20 min and washed twice with PBS. Subsequently, Vero cells were lysed with 0.1% Triton X-100 to count bacterial CFU in MHA plates (Liu et al., 2020).

### Drug Resistance Assay

*S. aureus* ATCC29213 was cultured in fresh MHB with 6-ES or oxacillin at concentrations of 0.5×MIC. After incubation at 37°C for 24 h, the bacterial suspensions were repassaged to new MHB for the next MIC assay.

### Molecular Docking

Discovery Studio 2020 was used to predict the possible binding mode of FtsZ, a-hemolysin, and 6-ES by the CDOCKER module of the receptor-ligand interaction section. The structures of FtsZ (Fujita et al., 2017) and *α*-hemolysin (Foletti et al., 2013) were used as receptors. The three-dimensional structure of 6-ES were prepared with ChemDraw, and then the conformation of ligand was calculated by docking study using CHARMm based docking tool in Discovery Studio 2020.

### Preparation of a Thermosensitive Hydrogel

Hydrogels were prepared with the cold solution method (Oliva et al., 2017). In brief, P407 was dissolved in PBS, and then the solutions were preserved at -20°C for 24 h to ensure complete dissolution. To prepare a 6-ES-loaded hydrogel (6-ES hydrogel), 6-ES was diluted in PEG400 and then added slowly into hydrogel solutions. The solution–hydrogel transition temperature (T_sol–hydrogel_) of the 6-ES hydrogel was measured.

### *In vivo* Skin Infection Model

Wounds were prepared in the backs of BALB/c mice and then infected with MRSA T144 (1 × 10^8^ CFU). Subsequently, wounds were topically administered 0.1 g of vehicle hydrogel, 0.1‰ 6-ES hydrogel, or 0.1‰ vancomycin hydrogel and then monitored for 10 days. At 5 days post infection, the bacterial burdens of MRSA in the wound were measured. All animal experiments were approved by the Institutional Animal Care and Use Committee of Qingdao Agricultural University.

### Statistical Analysis

Data are presented as the mean ± SD. Data were analyzed by analysis of variance (ANOVA) with GraphPad Prism seven to determine the least significant differences (*p* < 0.05).

## Results

### Alkaloids From *M. cordata* Exhibited Good Antibacterial Activity

**FIGURE 1:**
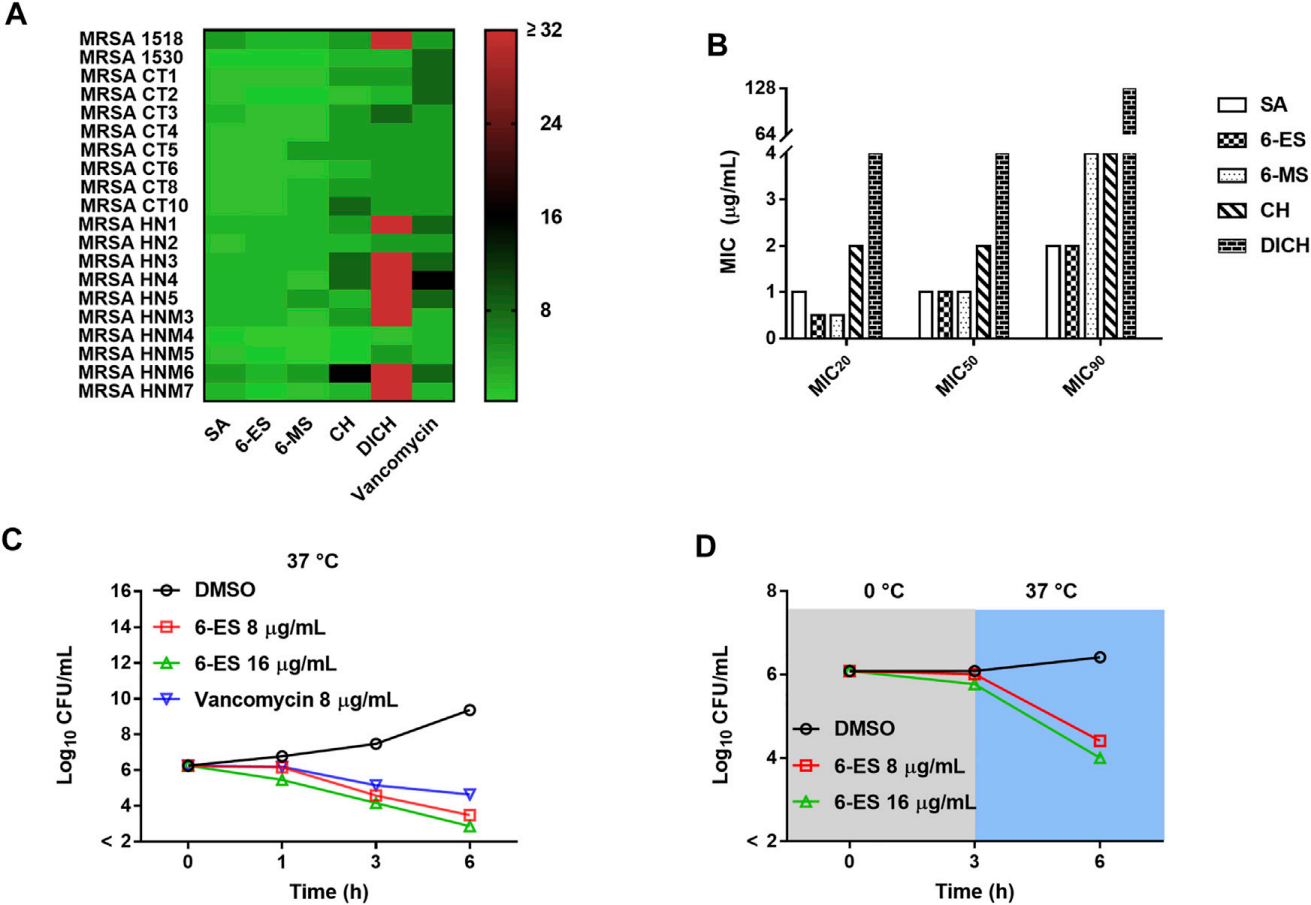
Antibacterial effects of SA, 6-ES, 6-MS, CH, and DICH against MRSA. **(A)** Heat map of the MICs of SA, 6-ES, 6-MS, CH, and DICH against MRSA strains. **(B)** MIC_20_, MIC_50_ and MIC_90_ of SA, 6-ES, 6-MS, CH, and DICH against MRSA strains. **(C)** MRSA T144 cells were cultured with 6-ES at 37°C, and the surviving bacteria were determined with the plate method at the indicated times. **(D)** Bactericidal activity of 6-ES against MRSA T144 at 0 and 37°C.

Alkaloids from *M. cordata* have been used to treat bacterial-associated diseases. Thus, the antibacterial effects of these alkaloids were investigated. Sanguinarine (SA), 6-ethoxysanguinarine (6-ES), and 6-methoxydihydrosanguinarine (6-MS), displayed good antibacterial activity against both Gram-positive bacteria and Gram-negative bacteria, whereas chelerythrine (CH) and dihydrochelerythrine (DICH) showed poor antibacterial effect against Gram-negative bacteria and some Gram-positive bacteria strains (Supplementary Table S1). Moreover, DICH showed better antibacterial effect against *S. aureus* than other bacterial strains. To investigate the structure-activity relationship of these alkaloids, the antibacterial activity of the alkaloids against clinically isolated MRSA strains was also investigated. The MIC_20_, MIC_50_, and MIC_90_ of SA, 6-ES, 6-MS, CH, and DICH against MRSA were 1/1/2, 0.5/1/2, 0.5/1/4, 2/2/4, and 4/4/128 μg/ml, respectively (Fig. 1AB). Additionally, 6-ES exhibited the best antibacterial activity against the MRSA strains. Structure-activity assay showed that the 6-ethoxy and 7,8-methylenedioxy modifications enhance the antibacterial activity against MRSA (Supplementary Figure S1).

### 6-Ethoxysanguinarine Exhibited Metabolism-dependent Bactericidal Action Against MRSA

To investigate the potential mode of action of 6-ES against MRSA, the bactericidal activity of 6-ES was investigated. Both 6-ES and vancomycin exhibited bactericidal activity against MRSA T144.6-ES at 8 μg/ml and 16 μg/ml killed MRSA T144 at 6 h, similar to vancomycin at 6 h (Figure 1C). To investigate whether the bacterial metabolic state affects the bactericidal effect of 6-ES, the bactericidal effect of 6-ES against MRSA T144 was investigated at 0 and 37°C. As shown in Figure 1D, 6-ES exhibited a lower bactericidal effect at 0°C than at 37°C (Figure 1D).

### 6-Ethoxysanguinarine Rapidly Disrupted Membrane Function and Induced the Accumulation of ROS in *S. aureus*

**FIGURE 2:**
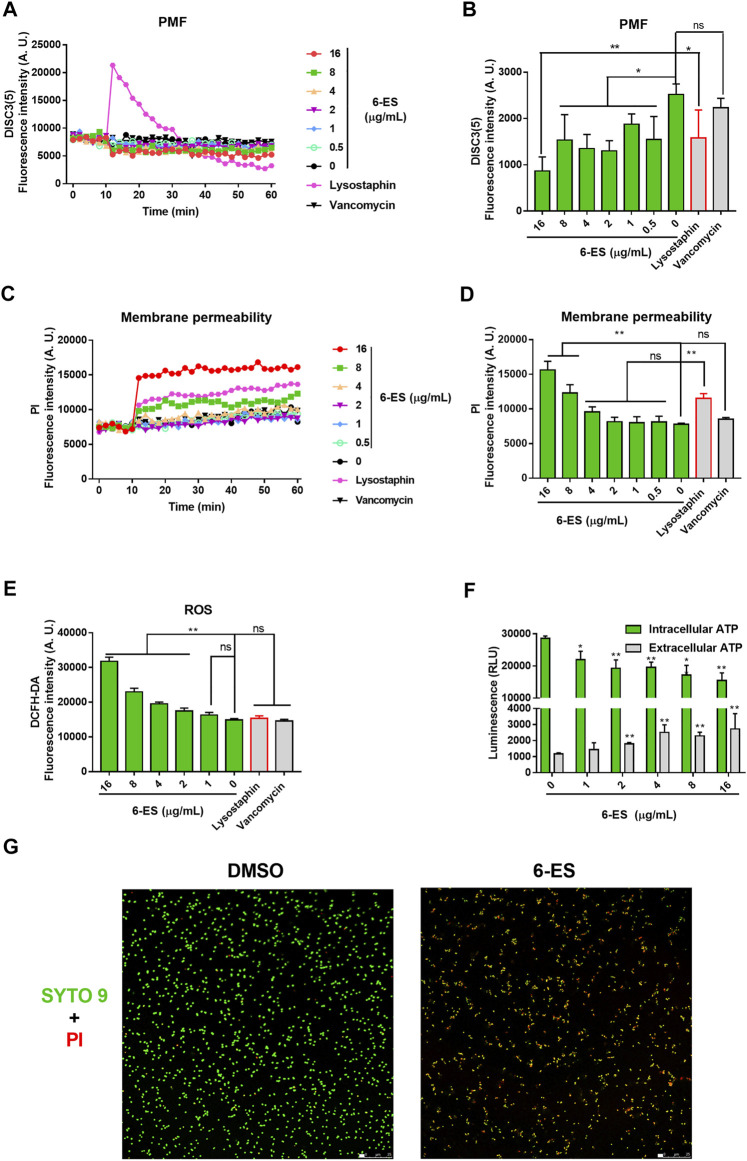
6-ES rapidly disrupted membrane function and promoted the generation of ROS in *S. aureus.* **(A, C)** MRSA T144 cells were incubated with DISC3 (5) or PI for 10 min and then treated with 6-ES for 50 min. The dynamic fluorescence intensities were measured every 2 min **(B,D)** DISC3 (5) or PI fluorescence intensities of MRSA T144 treated with 6-ES for 30 min. Data are presented as the means ± SDs. ^*^ *p* < 0.05, ^**^ *p* < 0.01. **(E)** MRSA T144 cells were incubated with DCFH-DA, followed by treatment with 6-ES for 30 min. The fluorescence intensities were measured at excitation/emission 488/525 nm. Data are presented as the means ± SDs. ^*^ *p* < 0.05, ^**^ *p* < 0.01. **(F)** MRSA T144 cells were incubated with 6-ES for 30 min, and the levels of extracellular ATP and intracellular ATP were measured. Data are presented as the means ± SDs. ^*^ *p* < 0.05, ^**^ *p* < 0.01. **(G)** MRSA T144 cells were incubated with 6-ES (16 μg/ml) or DMSO for 30 min and then stained with PI (red) and SYTO 9 (green).

**FIGURE 3:**
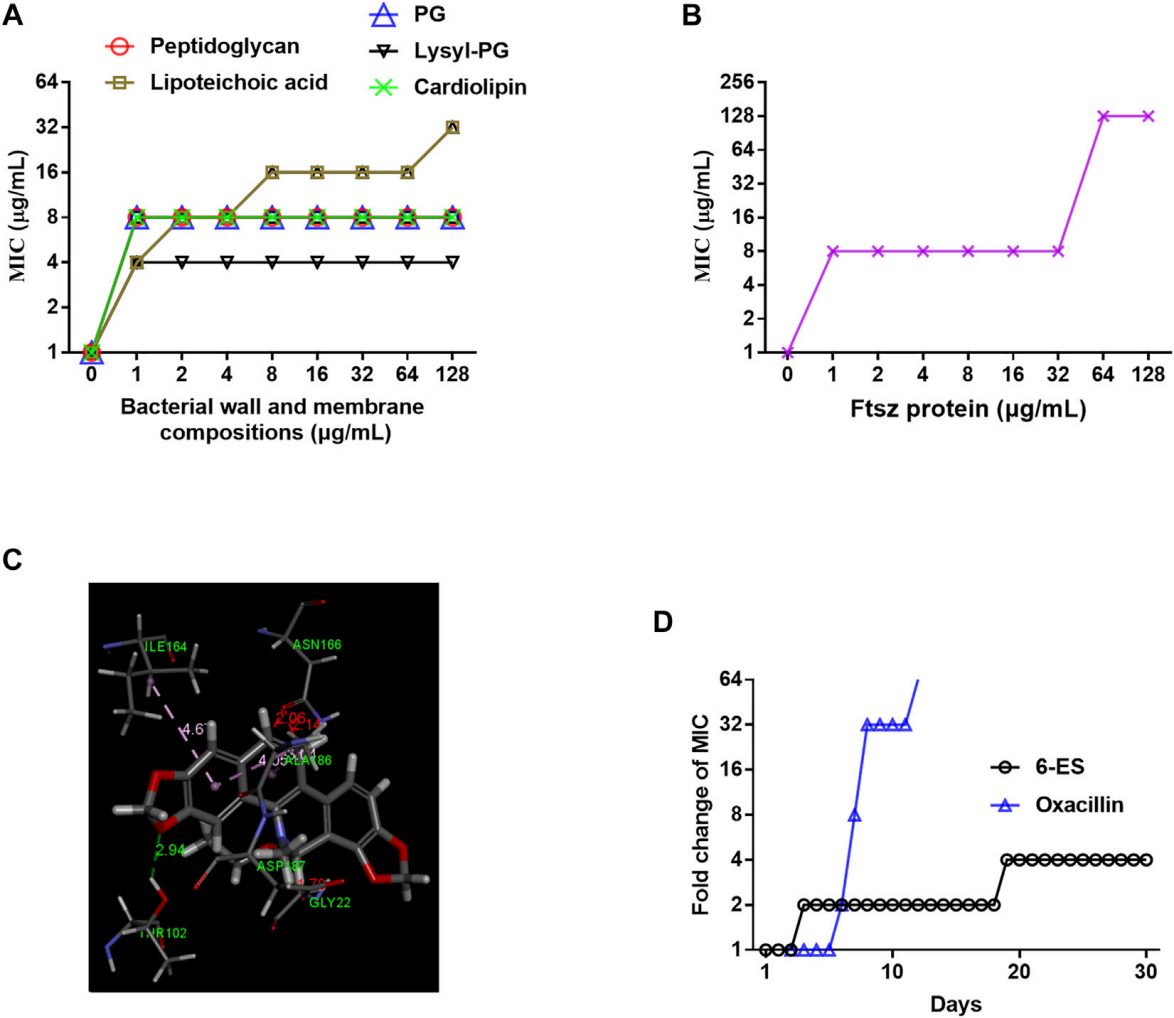
6-Ethoxysanguinarine inhibited bacterial growth possibly by targeting the bacterial wall and membrane and FtsZ. **(A,B)** The MIC of 6-ES against MRSA T144 in the presence of cell wall components, membrane phospholipids, and FtsZ. **(C)** Molecular docking of 6-ES with FtsZ. **(D)** 6-ES displayed a low level of resistance development. *S. aureus* ATCC29213 was continuously incubated with sub-MIC levels of 6-ES for 30 days. The sensitivity of bacteria to 6-ES was recorded.

To investigate the possible mode of action of 6-ES, the biochemical indexes of 6-ES-treated *S. aureus* were measured with different fluorescent probes. The redistribution fluorescent dye 3,3′-Dipropylthiadicarbocyanine iodide (DISC3 (5)) responds to bacteria membrane depolarisation or hyperpolarisation by membrane potential (Δψ)-dependent outflow from or uptake into the cells, reflected in changes in the fluorescence intensity. Upon treatment, 6-ES and lysostaphin induced rapid changes of DISC3 5) fluorescence intensity, whereas vancomycin treatment showed no effect on DISC3 5) fluorescence intensity (Figure 2A, B). These results suggested that 6-ES and lysostaphin rapidly depolarized Δψ of the proton motive force (PMF) in *S. aureus*. When bacteria cytoplasmic membrane is disrupted, the fluorescence intensity of PI increase after binding to DNA. Upon treatment, 6-ES and lysostaphin rapidly disrupted the membrane of *S. aureus*, as evidenced by a rapid increase in PI fluorescence (Figure 2C, D), consistent with the collapse of Δψ (Figure 3A, B). Moreover, treatment with 6-ES increased the extracellular ATP levels and decreased the intracellular ATP levels (Figure 2F), supporting the destruction of membrane function, as confirmed by the increased number of bacteria with disrupted membranes (red/yellow) (Figure 2G). DCFH-DA produce dichlorofluorescein with green fluorescence via intracellular esterase decomposition and ROS oxidant. Thus, DCFH-DA was used to detect intracellular ROS. 6-ES promoted the accumulation of ROS in *S. aureus*, suggesting that 6-ES triggered oxidative stress in *S. aureus* (Figure 2E). Moreover, SA, 6-ES and 6-MS displayed greater effects on membrane functions than CH and DICH (Supplementary Figure S2), consistent with the high antibacterial activities of SA, 6-ES and 6-MS against MRSA (Figure 1A, B). Collectively, 6-ES displayed antibacterial activities against MRSA, possibly *via* the disruption of membrane functions and the generation of ROS. Next, we tried to explain the structure−activity relationship of alkaloids on the membrane function of S. aureus. The addition of SA, 6-ES, 6-MS, CH, and DICH at 16 μg/ml disrupted the PMF, as evidenced by low DISC3(5) intensity after incubation for 50 min (Supplementary Figure S2). Interestingly, SA, 6-ES, and 6-MS significantly increased the intensity of PI and DCFH-DA, whereas CH and DICH did not significantly affect the intensity of propidium iodide (PI) and DCFH-DA (Supplementary Figure S2 CDEF), indicating 7,8-Methylenedioxy enhanced the antibacterial activity of alkaloids via increasing membrane permeability and promoting ROS generation. Additionally, the treatment with 6-ES induced higher fluorescence intensity of PI and DCFH-DA than that treated with SA (Supplementary Figure S2 CDEF).

### 6-Ethoxysanguinarine Modulated the Bacterial Metabolism Response

Membrane functions were inferred to lead to metabolic disorders, evidenced by low levels of intracellular ATP and the generation of ROS. Additionally, 6-ES suppressed MRSA T144 at sub-MIC levels. To clarify specific molecular mechanisms, we performed transcription analyses of MRSA T144 under treatment with sub-MIC 6-ES for 12 h. RNA-sequencing analysis showed an upregulation of 190 and downregulation of 142 differentially expressed genes (DEGs) in 6-ES-treated MRSA T144 (Supplementary Figure S3A). Gene ontology (GO) annotation analysis showed that these DEGs were correlated with cellular components (e.g., ribosome), molecular functions (e.g., structural constituent of ribosome) and biological processes (e.g., multiorganism cellular process) (Supplementary Figure. S3B). Kyoto Encyclopedia of Genes and Genomes (KEGG) enrichment analysis showed that downregulated DEGs were involved in the ribosome, *S. aureus* infection, and so on, while upregulated DEGs were involved in microbial metabolism in diverse environments, the TCA cycle, carbon metabolism and so on (Supplementary Figure S3C, D). To counter disturbances, bacteria always initiate responses to maintain cell homeostasis (Lister and Horswill, 2014). It is plausible that 6-ES modulated cell metabolism, including low levels of intracellular ATP, which was compensated by an upregulation of metabolism in diverse environments and TCA cycle-related genes. Interestingly, *S. aureu*s infection-associated genes were drastically downregulated, implying the weakened virulence of *S. aureus* by 6-ES (Supplementary Figure S3E).

### 6-Ethoxysanguinarine Inhibited Bacterial Growth Possibly by Targeting the Bacterial Membrane and FtsZ, With a Low Level of Resistance Development

Given that 6-ES rapidly interfered with bacterial membrane function, we hypothesized that the bacterial wall and membrane were potential targets of 6-ES. We sought to compare the antibacterial activity of 6-ES on peptidoglycan and lipoteichoic acids in the bacterial wall and phospholipids, including phosphatidylglycerol (PG), lysyl-phosphatidylglycerol (lysyl-PG), and cardiolipin (CAL) in the membrane of *S. aureus*. Interestingly, all these components of the bacterial wall and membrane inhibited the antibacterial activity of 6-ES against MRSA T144 (Figure 3A), indicating that 6-ES bound to the bacterial wall and membrane in of *S. aureus*. Based on the structural characteristics, membrane phospholipid were potential direct targets (Scheffers and Pinho, 2005). We hypothesized that 6-ES not only disrupts the intact membrane by targeting the bacterial membrane but also may have cytosolic targets. The Filamentous temperature-sensitive protein Z (FtsZ) protein is the bacterial homologue of tubulin that is essential for bacterial cell division and has been identified as a potential target of sanguinarine (Beuria et al., 2005; Ur Rahman et al., 2020). The inhibitory effect of FtsZ on the antibacterial effect of 6-ES supported that FtsZ was a potential target of 6-ES (Figure 3B). Molecular docking showed that 6-ES binds with FtsZ via interaction with ILE164, ASN166, ALA186, ASP187, GLY22, IAR10 through hydrogen bonding and electrostatic interactions (Figure 3C). Considering that the antibacterial action of 6-ES is a novel antibacterial mode of action, we sought to evaluate the resistant development of *S. aureus* in the presence of 6-ES for 30 days. At day 10, the MIC of oxacillin increased 32-fold, whereas the MIC of 6-ES increased 2-fold (Figure 3D), confirming that 6-ES displayed low levels of resistance development.

### 6-Ethoxysanguinarine Suppressed the Virulence of *S. aureus* and Modulated the Host Immune Response

**FIGURE 4:**
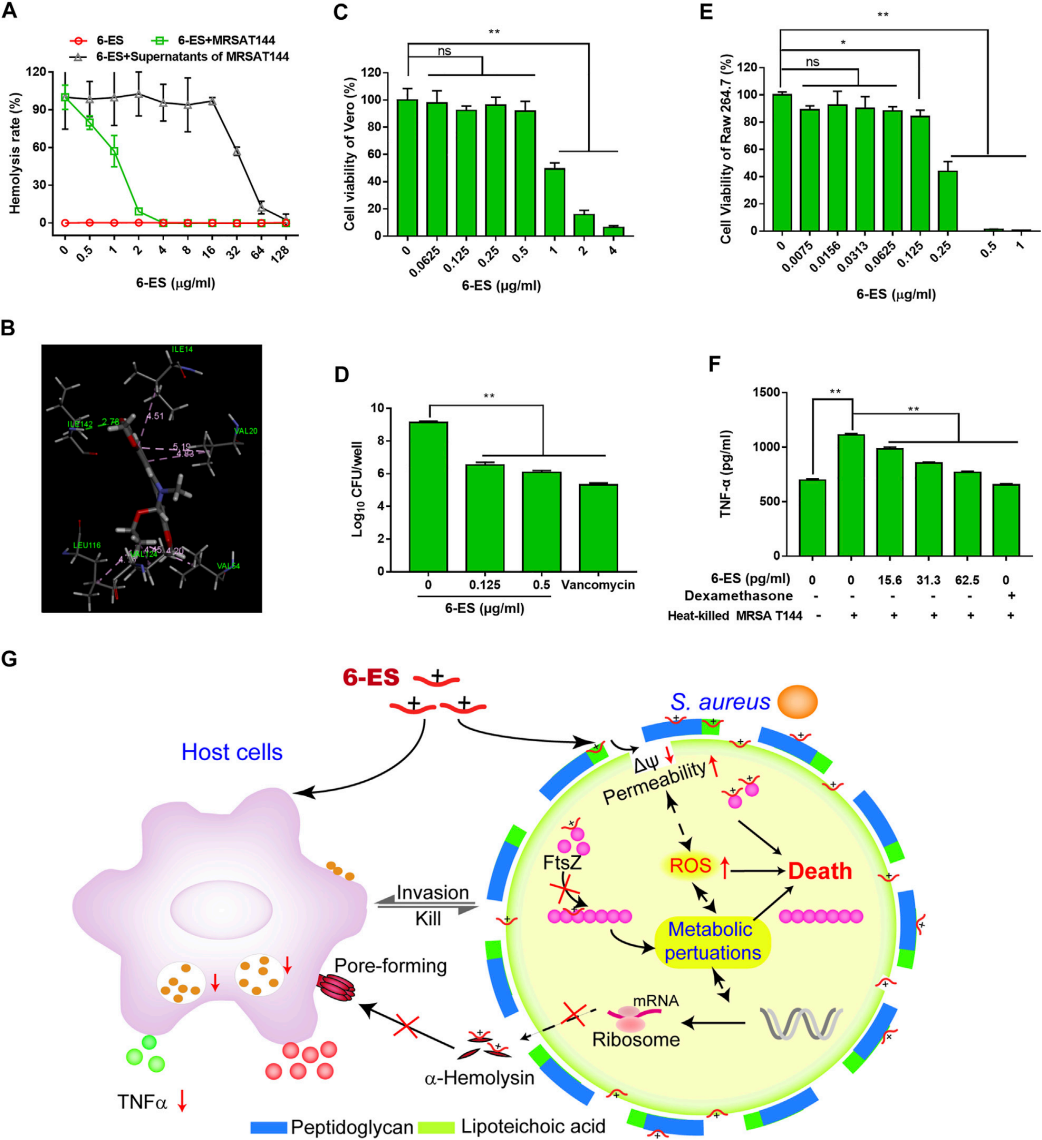
6-ES suppressed the virulence of *S. aureus* and modulated the host immune response. **(A)** MRSA T144 cells were cultured with different concentrations of 6-ES or DMSO for 12 h, and the supernatants were harvested to test the hemolytic activities toward sheep red blood cells. The supernatants from DMSO-treated MRSA T144 were used to evaluate the toxin neutralization effect of 6-ES against *α*-hemolysin. **(B)** Molecular docking of 6-ES with *α*-hemolysin. **(C, E)** Vero or RAW264.7 cells were treated with different concentrations of 6-ES for 24 h, and then, the cell viability was measured with the WST-1 method. **(D)** Vero cells were infected with MRSA T144 and then treated with 6-ES or vancomycin for 6 h. The intracellular bacterial number was measured by the plate method. **(F)** RAW264.7 cells were incubated with 6-ES for 1 h and then stimulated with heat-killed MRSA T144 for 24 h. After incubation, the supernatants were harvested to detect the level of TNFα by ELISA. **(G)** Multiple mechanisms for 6-ES activity against MRSA. 6-ES collapsed the membrane potential, increased membrane permeability, and promoted the accumulation of ROS in *S. aureus*, possibly by targeting the membrane and FtsZ, which led to metabolic disorders and cell death of *S. aureus*. Moreover, 6-ES modulated metabolic processes, such as cell division and virulence factors. 6-ES directly suppressed the hemolytic activity induced by *α*-hemolysin. In addition, 6-ES modulated the host inflammatory response caused by *S. aureus*. Multiple mechanisms of 6-ES supported 6-ES as a novel leading compound with antibacterial activity, antivirulence activity, and host immunomodulatory activity.

Since *α*-hemolysin is an important virulence factor of *S. aureus*, we sought to evaluate the antivirulence effect of 6-ES with sheep red blood cells and the supernatants of *S. aureus*. As shown in Figure 4A, 6-ES at 0.5–128 μg/ml had no hemolytic toxicity and suppressed the hemolytic toxicity of the supernatants of 6-ES-treated *S. aureus* (Figure 4A). Importantly, 6-ES directly inhibited the hemolytic toxicity of the supernatants of MRSA T144 (Figure 4A). Molecular docking showed that 6-ES interacted with ILE142, ASN14, VAL20, LEU116, VAL124, VAL54 of a-hemolysin via pi-alkyl hydrogen bonds (Figure 4B). Next, to evaluate the protective efficacy of 6-ES in a MRSA-Vero cell infection model, the cytotoxicity of 6-ES to Vero cells was first investigated. We found that 6-ES at 0.125–0.5 μg/ml showed no toxicity to Vero cells (Figure 4C). Thus, doses of 0.125–0.5 μg/ml were applied to assess the efficacy of 6-ES in the MRSA-Vero cell infection model. Treatment with 6-ES at safe doses of 0.125 and 0.5 μg/ml significantly decreased the number of intracellular MRSA T144 cells, suggesting that 6-ES was efficacious in eliminating intracellular MRSA (Figure 4D). In addition, we next explored whether 6-ES possesses immunomodulatory activity similar to that of sanguinarine (Meng et al., 2018). 6-ES at a safe dose suppressed the production of TNFα by MRSA T144-stimulated RAW264.7 cells (Fig. 4EF), indicating that 6-ES suppressed the inflammatory response caused by MRSA. Overall, the results showed that 6-ES protected against MRSA infections *via* multiple mechanisms, such as antivirulence, host immune response, and antibacterial activity (Figure 4G; Supplementary Figure S5).

### A 6-Ethoxysanguinarine-loaded Thermosensitive Hydrogel Promoted the Wound Healing of Skin Infected With MRSA

**FIGURE 5:**
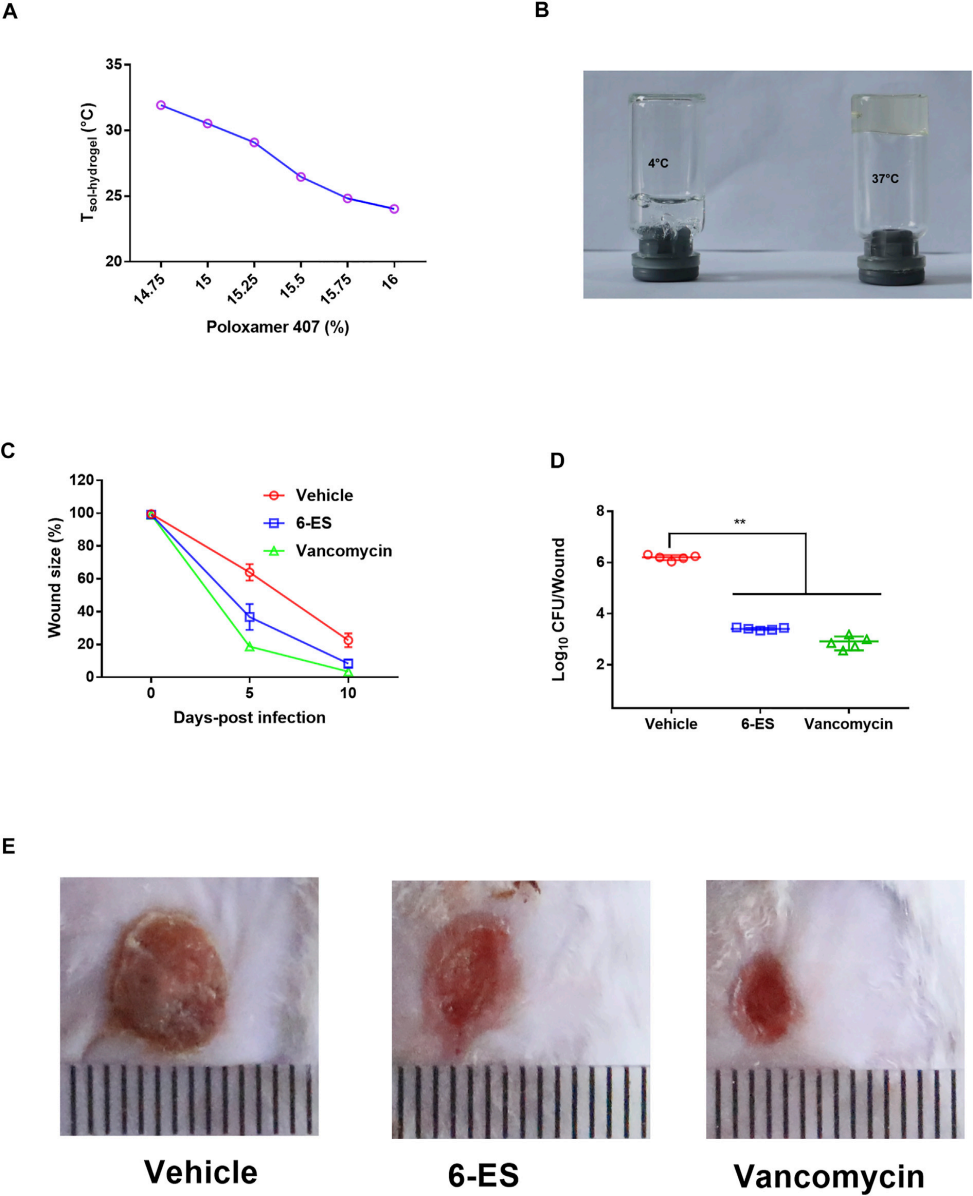
6-ES hydrogel promoted wound healing in mice infected with MRSA. **(A)** T_sol–hydrogel_ of 6-ES-loaded hydrogel with different concentrations of P407. **(B)** Digital pictures of 6-ES hydrogel at 4 and 37°C. **(C–E)** Wound sizes, bacterial burdens, and images of MRSA T144-infected wounds in mice treated with 6-ES hydrogel, vancomycin hydrogel, or vehicle hydrogel.

To screen the preparation of 6-ES hydrogels, the T_sol–hydrogel_ of 6-ES hydrogels with different concentrations of P407 was first investigated. The T_sol–hydrogel_ of the 6-ES hydrogel decreased as the concentrations of P407 increased (Figure 5A). 6-ES hydrogels containing 15% P407, 5% PEG400, and 0.1‰ 6-ES were used with a T_sol–hydrogel_ at 30.5°C (Figures 5A, B). A skin infection model was used to assess the *in vivo* antibacterial efficacy of the 6-ES hydrogel. The 6-ES hydrogel and vancomycin hydrogel promoted skin wound healing in mice infected with MRSA (Figures 5C, E). The wounds treated with the 6-ES hydrogel and vancomycin hydrogel exhibited lower bacterial burdens than those in the control hydrogel group (Figure 5D). These results suggest that the 6-ES-loaded P407 hydrogel is a potential drug candidate for the treatment of MRSA-associated skin infections.

## Discussion

Alkaloids from *M. cordata* have been used to treat bacterial-associated diseases (Xue et al., 2017). In this study, we found that 6-ES displayed high activity against MRSA possible via interfering membrane and metabolism functions. 6-ES also inhibited the hemolytic activity of *α*-hemolysin, and alleviated inflammatory responses caused by MRSA. Moreover, 6-ES protected against MRSA in both Vero cells model and mice skin model. These results demonstrated that 6-ES from M. cordata is one potential leading compound with antibacterial, anti-virulence, and host modulation activity for the treatment of MRSA associated infection.

Bactericidal assay of 6-ES supported that 6-ES was one bactericidal agent (Figure 1C). Death from most bactericidal antibiotics is associated with membrane functions, such as cellular respiration, proton motive force (PMF), adenosine triphosphate (ATP) synthesis, ROS generation (Magnowska et al., 2019). In our study, we found that 6-ES inferenced member functions of *S. aureus* (Figure 2; Supplementary Figure S2), supported that 6-ES was one membrane-active antibacterial agents. Membrane functions were inferred to lead to metabolic disorders, evidenced by low levels of intracellular ATP and the generation of ROS (Figures 2E, F; Supplementary Figures S2E, F). Moreover, the 6-ethoxy and 7,8-methylenedioxy groups promoted the increase in membrane permeability and ROS generation, supporting that 6-ethoxy and 7,8-methylenedioxy modifications enhance the antibacterial activity against MRSA (Figure 2; Supplementary Figures S1, 2). The metabolic state of bacteria has been shown to affect antibiotic efficacy (Lopatkin et al., 2019). The bactericidal activity of 6-ES was partly dependent on the metabolic state, with implications for its potential effect on bacterial metabolism (Figure 1D). Transcription analyses supported 6-ES modulated cell metabolism evidence by the modulation of metabolism diverse environments, TCA cycle, *S. aureu*s infection-associated genes, and so on (Supplementary Figure S3). Inhibition assays and growth assay provided compelling evidence that the bacterial membrane and FtsZ were potential targets of 6-ES, confirming that alkaloids such as 6-ES and SA were a novel membrane-active antibacterial agent (Figures 3A, B, Supplemntary Figure S4). The novel mode of action of 6-ES represents one new type of antibacterial agent to avoid resistance development (Figure 3D).

*S. aureus* can invade and replicate within many types of host cells to escape clearance by host immune defense or antibiotic killing (Bravo-Santano et al., 2019; Tribelli et al., 2020). *α*-hemolysin can help the evasion of *S. aureus* from the host response and leads to the death of host cells (Putra et al., 2019). Studies have showed that chalcone and myricetin directly inhibited the hemolytic toxicity of *α*-hemolysin (Zhang et al., 2017; Wang et al., 2020). Interestingly, the inhibitory effect of 6-ES on the hemolytic toxicity of the supernatants of MRSA T144, indicating that *α*-hemolysin was a direct target of 6-ES. The inflammatory response contributes to host damage caused by pathogens (Kay et al., 2019). Moreover, studies have showed that sanguinarine exhibited anti-inflammatory effects (Li et al., 2021; Wang et al., 2021). Interestingly, the inhibitory effect of 6-ES on the inflammatory response in MRSA-stimulated Raw264.7 cells supported that 6-ES also exhibited anti-inflammatory effects. Interestingly, 6-ES was efficacious in eliminating intracellular MRSA (Figure 4D), consistent with the down regulation of *S. aureu*s infection-associated genes (Supplementary Figure S3). These results supported that 6-ES was one novel antibacterial agent with antivirulence activity, and host immunomodulatory modulation activity.

Studies have showed alkaloids such as sanguinarine had hepatotoxic, cytotoxicity, cardiotoxicity, mutagenicity, carcinogenicity, genotoxicity effects, and so on (Singh and Sharma, 2018). Although 6-ES exhibited protective effect against MRSA in MRSA-Vero model (Figure 4D), the application of 6-ES for systemic infection should be limited due to its cytotoxicity on Vero cells and RAW264.7 cells (Fig. 4CE). Moreover, Studies have showed that alkaloids from M. cordata displayed toxicity *in vivo*, such as cardiotoxicity, hepatotoxicity, and so on (Rad et al., 2017). S. aureus is one major pathogen that caused skin infection. Topical administration for a localized infection can avoid the side effects of systemic applications (Pitorre et al., 2021). P407-based thermosensitive hydrogels have been widely used as vehicles of many drugs for topical delivery (Liu et al., 2019; Cristiano et al., 2020). The protective efficacy of 6-ES-loaded P407 hydrogel on MRSA skin infection model supported its use as a candidate for the prevention of *S. aureus* associated skin infection (Figure 5). Furthermore, its potential toxicity and structural optimization remain to be addressed.

In conclusion, the alkaloid 6-ES displayed good antibacterial activity against MRSA possibly *via* interfering with membrane and metabolism functions by targeting the membrane and FtsZ. 6-ES also directly suppressed the hemolytic activity of *α*-hemolysin, alleviated inflammatory responses, and eliminated intracellular MRSA *in vitro*. Moreover, the 6-ES-loaded hydrogel promoted wound healing and elimination of bacteria in mice infected with MRSA. All these results supported alkaloids from *Macleaya cordata* including 6-ES as novel potential antibacterial candidates and leading compounds with antibacterial activity, antivirulence activity, and host immunomodulatory activity (Supplementary Figure S5).

## Data Availability

The original contributions presented in the study are included in the article/Supplementary Material, further inquiries can be directed to the corresponding author.
