# Supplementary material for: Natural Antibacterial and Antivirulence Alkaloids From Macleaya cordata Against Methicillin-Resistant Staphylococcus aureus
Source: Front Pharmacol. 2022 Mar 17;13:813172. doi: 10.3389/fphar.2022.813172 (PMC8968424; doi:10.3389/fphar.2022.813172)
Supplement: Supplementary file 1 [file DataSheet1.doc]

**Supplementary material**

**Natural antibacterial and antivirulence alkaloids from *Macleaya cordata* against methicillin-resistant *Staphylococcus aureus***

Zhi-Hai Liu^ab1^, Wei-Mei Wang^a1^, Zhen Zhang ^a1^, Liang Sun ^a1^, Shuai-Cheng Wu^ac*^

a College of Veterinary Medicine, Qingdao Agricultural University, No.700 Changcheng Road, Qingdao, Shandong 266109, China.

b College of Chemistry and Pharmaceutical Sciences, Qingdao Agricultural University, No.700 Changcheng Road, Qingdao, Shandong 266109, China.

c Beijing Advanced Innovation Center for Food Nutrition and Human Health, College of Veterinary Medicine, China Agricultural University, No.2 Yuanmingyuan West Road, Beijing 100193, China.

1 Contribute equally.

* Corresponding authors:

Shuai-Cheng Wu, Tel.: +86+19560727869. Fax.:+86+053286080129

E-mail: wushuaicheng10@163.com

**Table**

**Table S1. MIC of alkaloids from *Macleaya cordata* (μg/ml)**

| **Strains** | **SA** | **6-ES** | **6-MS** | **CH** | **DICH** | **Antibiotics** |
| --- | --- | --- | --- | --- | --- | --- |
| **Gram-positive** |  |  |  |  |  | Vancomycin |
| MSSA ATCC 29123 | 1 | 4 | 4 | 8 | 32 | 2 |
| MRSA T144 | 2 | 1 | 2 | 2 | 2 | 2 |
| *B. subtilis* ATCC 6051 | 1 | 1 | 0.5 | 1 | 128 | 0.25 |
| *E. faecalis* VRE1010798 | 8 | 8 | 16 | 32 | >128 | >128 |
| **Gram-negative** |  |  |  |  |  | Colistin B |
| *E. coli* ATCC25922 | 4 | 8 | 8 | 128 | ＞128 | 1 |
| *E. coli* B2 | 16 | 16 | 16 | 32 | ＞128 | 8 |
| *P. aeruginosa* 14 | 16 | 16 | 16 | 32 | ＞128 | 1 |
| *K. pneumonia* ATCC43816 | 16 | 8 | 16 | 64 | ＞128 | 16 |


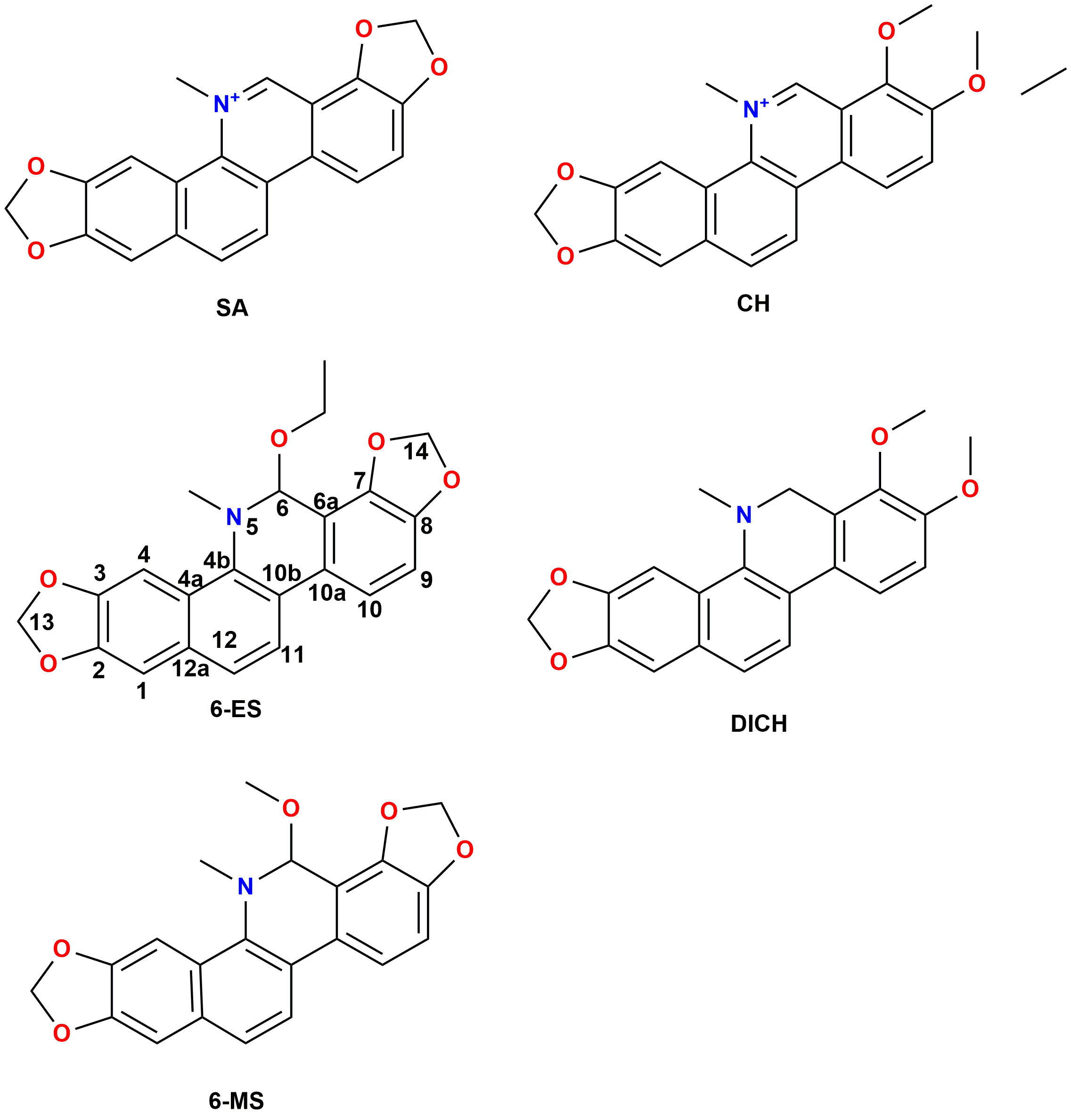


**Figure S1.** Chemical structures of alkaloids from M. cordata.


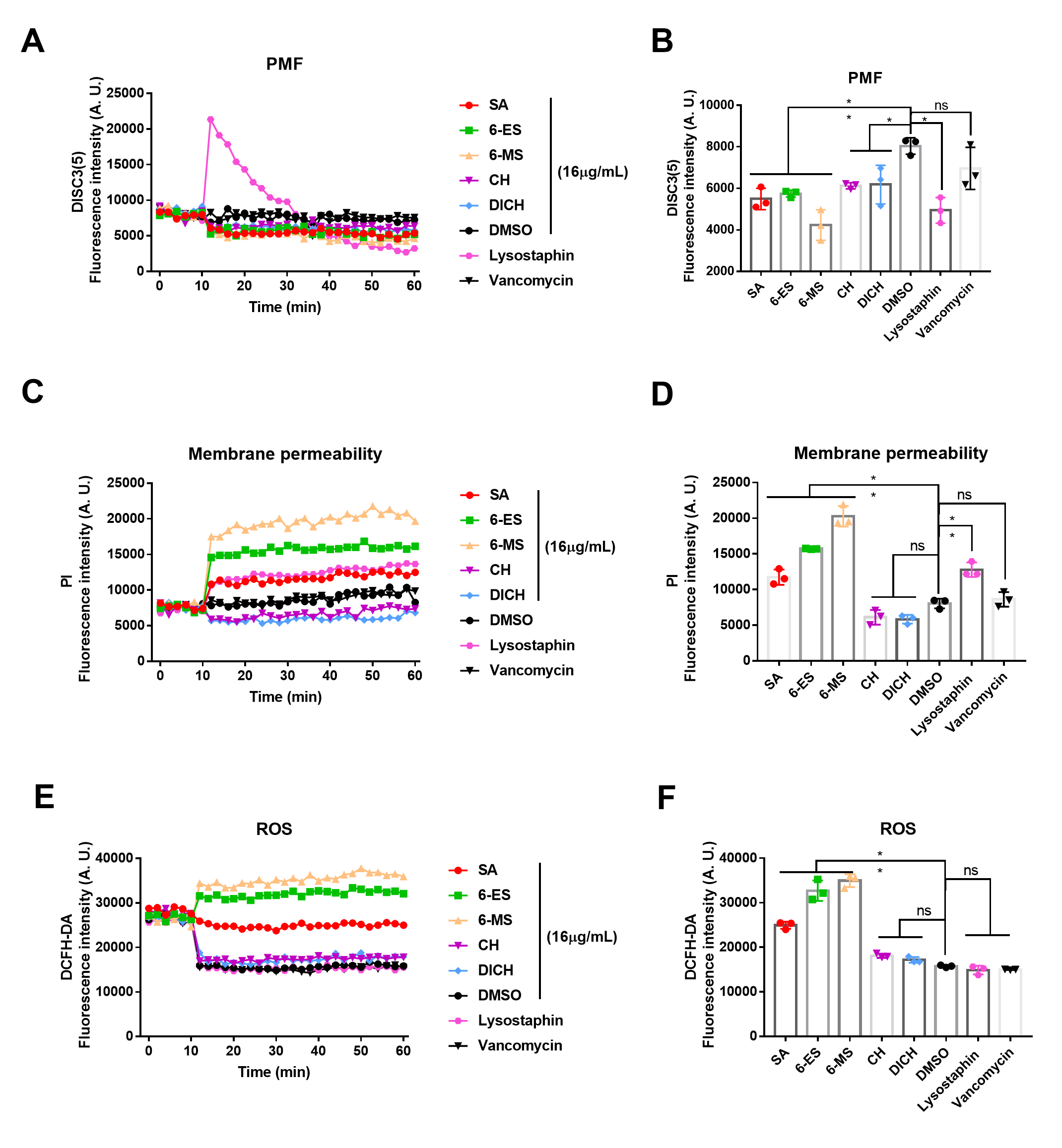


**Figure S2.** Structure-activity relationship of 6-ES on the membrane function of MRSA.

(A, C, E) MRSA T144 cells were incubated with DISC3(5), PI, or DCFH-DA for 10 min and then treated with SA, 6-ES, 6-MS, CH, or DICH for 50 min. The dynamic fluorescence intensities were measured every 2 min. (B, D, F) The fluorescence intensities of MRSA T144 treated with SA, 6-ES, 6-MS, CH, or DICH for 30 min. Data are presented as the means ± SDs. ^*^*P* < 0.05, ^**^ *P* < 0.01.

**METHODS**

**Transcriptome analysis**

MRSA T144 cells at the exponential phase were cultured with 6-ES at 0.5×MIC in MHB for 12 h, and then the bacteria were harvested by centrifugation for extraction of RNA. RNA was extracted using an EASYspin Plus Kit (Aidlab, Beijing, China) and sequenced using a HiSeq 4000 platform with a paired-end read length of 150 bp.

**Microbial growth curve analysis**

Growth curve analysis was carried out by measuring the absorbance at 600 nm. MRSA T144 cells were adjusted to approximately 1*10^6^ CFU/ml and were then cultured with 6-ES in 96-well plates for 24 h at 37 °C. The OD600 value was measured using an enzyme-linked immunosorbent assay reader.

**RESULTS**

**6-Ethoxysanguinarine modulated the bacterial metabolism response**

RNA-sequencing analysis showed an upregulation of 190 and downregulation of 142 differentially expressed genes (DEGs) in 6-ES-treated MRSA T144 (Fig. S3A). Gene ontology (GO) annotation analysis showed that these DEGs are correlated with cellular components (e.g., ribosome), molecular functions (e.g., structural constituent of ribosome) and biological processes (e.g., multiorganism cellular process) (Fig. S3B). Kyoto Encyclopedia of Genes and Genomes (KEGG) enrichment analysis showed that downregulated DEGs were involved in the ribosome, *S. aureus* infection, and so on, while upregulated DEGs were involved in microbial metabolism in diverse environments, the TCA cycle, carbon metabolism and so on (Fig. S3CD). To counter disturbances, bacteria always initiate responses to maintain cell homeostasis. It is plausible that 6-ES modulated cell metabolism, including low levels of intracellular ATP, which was compensated by an upregulation of metabolism in diverse environments and TCA cycle-related genes. Interestingly, *S. aureu*s infection-associated genes were drastically downregulated, implying the weakened virulence of *S. aureus* by 6-ES (Fig. S3E).


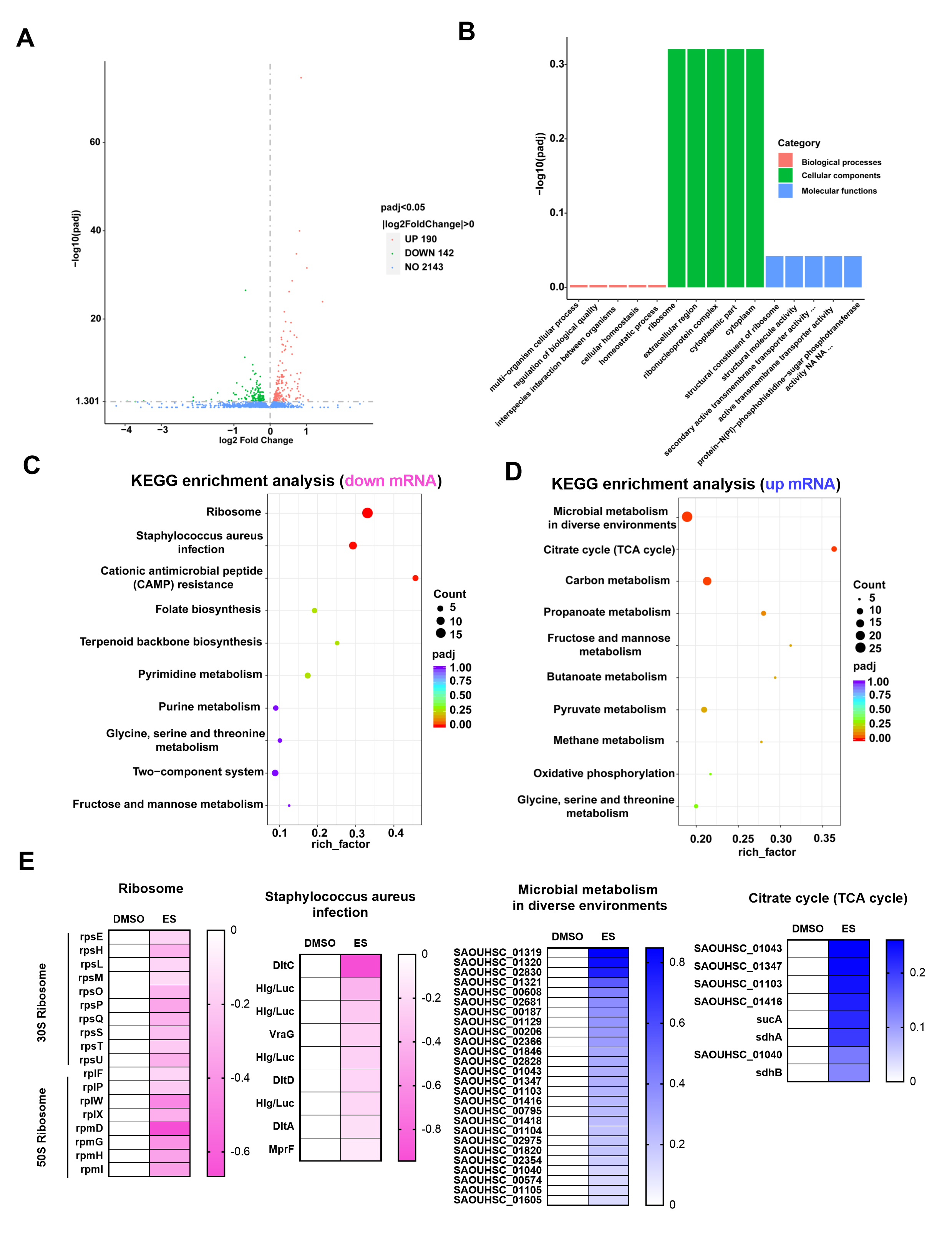


**Figure S3.** Transcriptome analysis of MRSA T144 after exposure to 6-ethoxysanguinarine.

(A) Volcano plot, (B) GO annotation, and (CD) KEGG enrichment analysis of the differentially expressed genes (DEGs) in MRSA T144 treated with 6-ES (0.5 μg/ml) for 12 h. The x- and y-axes in (A) represent the expression changes and corresponding statistically significant degree, respectively. An adjusted padj < 0.05 and log2 fold change ≥1 were applied as the cutoff for significant DEGs. (E) Select differentially expressed genes.

**6-ES inhibited the growth of MRSA T144**

As shown in Fig. S4, 6-ES at the concentrations of 0.12--2 μg/ml inhibited the growth of MRSA T144, whereas 6-ES at the concentrations of 0.03-0.06 μg/ml did not affect the growth of MRSA T144.


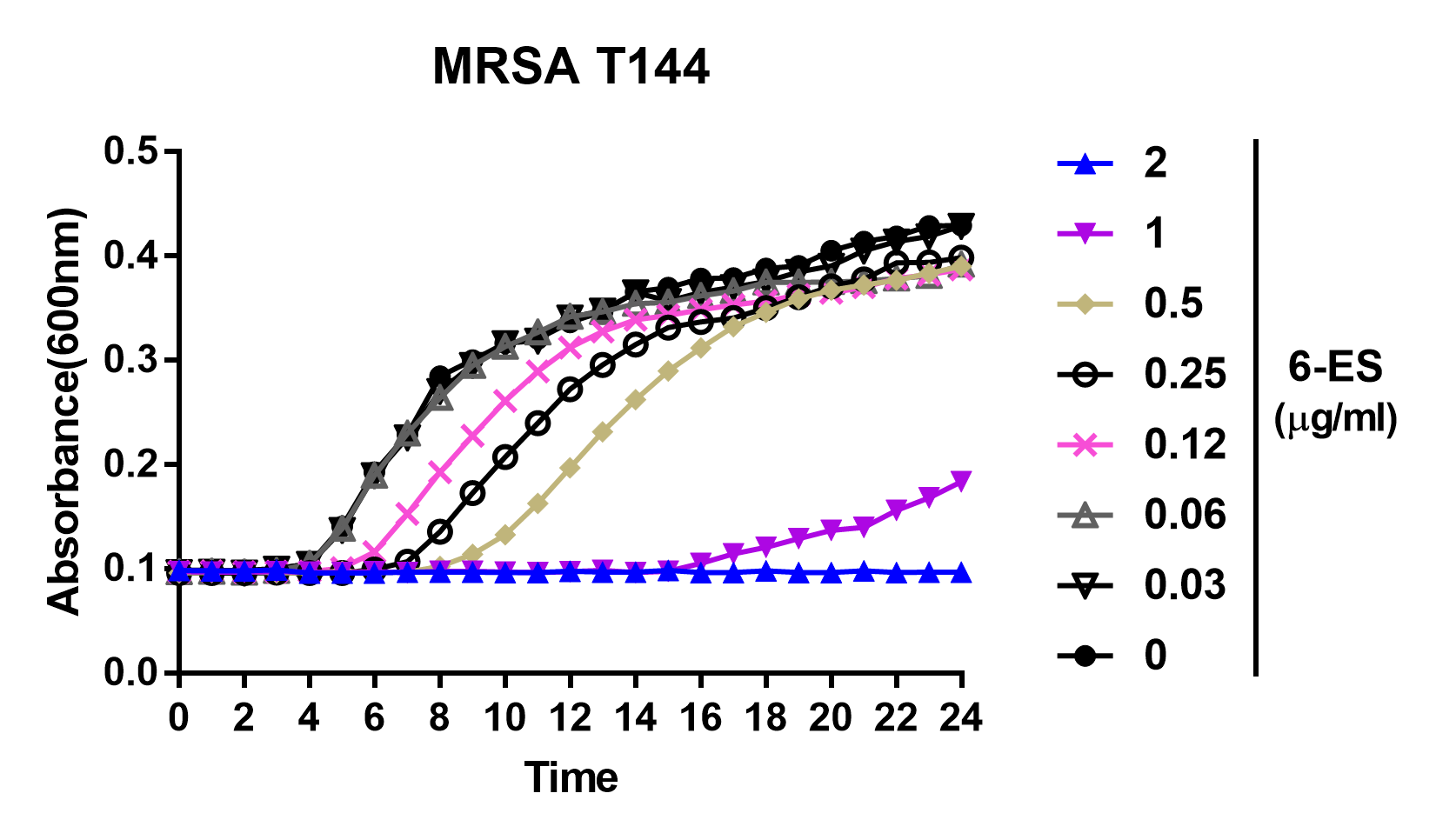


**Figure S4. Growth curve of MRSA treated with 6-ES.**

MRSA T144 cells were cultured with different concentrations of 6-ES for 24 h, and the OD value at 600 nm was measured with an ELISA reader.
